# P300 Wave Alterations and Cognitive Impairment in Cerebellum Lesions

**DOI:** 10.1007/s12311-023-01570-0

**Published:** 2023-05-26

**Authors:** Sourav Nanda, José Lapeña-Motilva, Amar Kumar Misra, Gautam Guha, Sinjan Ghosh, Akash Manna, Soumit Roy, Julián Benito-León

**Affiliations:** 1 Department of Neurology, N.R.S. Medical College and Hospital, Kolkata, India; 2 Department of Neurology, University Hospital “12 de Octubre’’, Madrid, Spain; 3 Department of Neurology, Baycrest Hospital, Toronto, ON Canada; 4 Department of Community Medicine, IQ City Medical College, Durgapur, West Bengal India; 5 Research Institute (I+12), University Hospital “12 de Octubre’’, Madrid, Spain; 6 Centro de Investigación Biomédica en Red Sobre Enfermedades Neurodegenerativas (CIBERNED), Madrid, Spain; 7 Department of Medicine, Complutense University, Madrid, Spain

**Keywords:** P300 Wave, Cognitive Performance, Cerebellum

## Abstract

Patients with cognitive deficits have a prolonged latency and reduced amplitude of the P300 wave. However, no study has correlated P300 wave alterations with the cognitive performance of patients with cerebellar lesions. We aimed to determine if the cognitive status of these patients was associated with P300 wave alterations. We recruited 30 patients with cerebellar lesions from the wards of the N.R.S. Medical College, Kolkata, in West Bengal (India). The Kolkata Cognitive Screening Battery tasks and the Frontal Assessment Battery (FAB) were used to assess the cognitive status and the International Cooperative Ataxia Rating Scale (ICARS) for cerebellar signs. We compared the results with the normative data of the Indian population. Patients had P300 wave alterations with a significant increase in latency and a non-significant trend in amplitude. In a multivariate model, P300 wave latency was positively associated with the ICARS kinetic subscale (*p* = 0.005) and age (*p* = 0.009), regardless of sex and years of education. In the model that included cognitive variables, P300 wave latency was negatively associated with performance in phonemic fluency (*p* = 0.035) and construction (*p* = 0.009). Furthermore, P300 wave amplitude was positively associated with the FAB total score (*p* < 0.001). In closing, patients with cerebellar lesions had an increase in latency and a decrease in the amplitude of the P300 wave. These P300 wave alterations were also associated with worse cognitive performance and some of the subscales of the ICARS, reinforcing that the cerebellum has motor, cognitive, and affective functions.

## Introduction

At least five large-scale networks involved in human cognition can be identified in the human brain: [1] (1) Dorsal frontoparietal network for spatial orientation—right hemisphere dominant with epicenters in the posterior parietal cortex, the frontal eye fields, and the cingulate gyrus; (2) Perisylvian language network—left hemisphere dominant with epicenters in Wernicke's and Broca's areas; (3) Limbic memory and emotion network with epicenters in the hippocampal-entorhinal regions and the amygdaloid complex; (4) Prefrontal network for executive function with epicenters in the lateral prefrontal cortex, orbitofrontal cortex, and posterior parietal cortex; and (5) Ventral occipitotemporal network for face and object recognition with epicenters in lateral temporal and temporopolar cortices. Some evidence suggests that the cerebellum is involved in the convergence of several of those networks [2].

Since the early nineteenth century, [3] the motor function of the cerebellum was discovered in pigeons when it was seen that those with cerebellar lesions presented difficulties in flight due to motor dyscoordination rather than weakness. Years later, cognitive and emotional alterations were first described in a patient with cerebellar agenesis [4]. Despite this, for the next 200 years, practically, only the cerebellum's motor function was considered significant [5].

Although reports were referring to the relevance of the cerebellum for language [6] or learning [7], the first studies establishing the cerebellar cognitive affective syndrome (also called Schmahmann syndrome) [8, 9] were carried out in the late twentieth century. In these studies [8, 9], clinically relevant deficits in executive function, visuospatial performance, linguistic processing, and mood dysregulation were reported in patients with cerebellum lesions. These findings have been replicated in patients belonging to different ages and pathologies- congenital, hereditary, injurious, or degenerative [5, 10]

Event-related potentials (ERP) have often been used to assess cognitive impairment [11]. These evoked potentials depend on the subject's selective attention to external stimuli [11]. Several components of ERPs have been identified, but of all of them, P300 or P3 wave has been the most consistent response [11]. So, most clinical studies focused on this P300 wave elicited from an experimental design called 'the oddball paradigm [11]. The subject is presented with two stimuli, one frequently occurring (the frequent stimulus) and the other infrequently (the rare stimulus) [11]. The P300 wave appears as a long-latency response to the rare auditory stimulus [11]. It has a latency-to-peak of 300 to 400 ms following the onset of the stimulus, its polarity is positive, and the maximal amplitude can be found in the midline of the central and parietal regions [11]. Patients with cognitive deficits have a prolonged latency and reduced amplitude of the P300 wave [12–15].

The effect of the P300 wave in spinocerebellar ataxia type 2 (SCA2) [16] and olivopontocerebellar atrophy [17] patients have been previously studied. An increase in P300 wave latency was found in both studies, [16, 17] but no change in amplitude in the first of them, [16] which was the only one that showed the amplitude results. In addition, clinical case reports of cerebellar stroke patients have shown alterations in the P300 wave with increased latency [18] or decreased amplitude [18, 19]. In both studies, [18, 19] these findings were related to the involvement of the cerebellum with attentional networks.

To our knowledge, no study has correlated P300 wave alterations with the cognitive performance of patients with cerebellar lesions. We aimed to determine if the cognitive status in patients with cerebellar lesions was associated with P300 wave alterations.

## Methods

Patients who came with a cerebellar disease (the diagnosis of cerebellar disease was performed by clinical evaluation and neuroimaging) with a good state of consciousness and who could consent at the time of the study were included. Those who had a personal history of cranial injuries, alterations of the sense organs, psychiatric disease, diagnosed or suspected dementia, central nervous system infections, traumatic brain injuries, increased intracranial pressure, alcoholism or other drug use, or that at the time of evaluation had sepsis or hepatic, renal, and pulmonary comorbidities, were excluded. Illiteracy was also an exclusion criterion. To select patients with lesions restricted to the cerebellum and not to any other part of the brain, magnetic resonance imaging (MRI) and 18F-fluorodeoxyglucose positron emission tomography-computed tomography (18F-FDG PET-CT) were performed. All patients had cerebellar hypometabolism on 18F-FDG PET-CT, not affecting any other part of the brain.

We recruited 30 patients who fulfilled the inclusion criteria from the wards of the N.R.S. Medical College and Hospital, Kolkata, in West Bengal (India), between May 1, 2019, and October 1, 2020.

The etiologies of the cerebellar lesions were the following: inherited neurodegenerative and other neurodegenerative disorders (*N* = 18), stroke (*N* = 4), following cerebellar tumor surgery (*N* = 3), myelin oligodendrocyte glycoprotein (MOG) antibody disease restricted to the cerebellum (*N* = 2), immune-mediated disorders (*N* = 2), and paraneoplastic disorders (*N* = 1).

The patients and their caregivers gave a detailed history of the current illness. General and neurological examination was performed on the subjects. Cerebellar signs were scored according to the International Cooperative Ataxia Rating Scale (ICARS) [20]. ICARS sub-scores were calculated for the following domains: posture and gait, kinetic functions, eye movement, and speech. The Kolkata Cognitive Screening Battery tasks and the Frontal Assessment Battery (FAB) were used to assess cognitive status [21] and cognitive abilities like conceptualization, mental flexibility, programming, inhibitory control, and environmental autonomy [22, 23]. Laboratory tests were performed to rule out other possible causes of cognitive decline (blood cell count, liver, renal, thyroid function tests, electrolytes, HIV, VDRL, anti-nuclear antibodies, and vitamin B12).

Auditory oddball paradigms were given to subjects in a sound-attenuated room using the NEUROSTIM software. One hundred auditory stimuli were presented binaurally at an approximately 80 dB sound pressure level. Standard stimuli (80% of probability) consisted of 500 Hz tones, whereas target stimuli (20% of probability) were 1000 Hz. Responses were recorded from the Cz electrode at the vertex referenced to mastoids. We compared the results with the normative data of the Indian population [24].

### Statistical Analyses

The data were analyzed using Python 3.9.7 and the packages pandas 1.3.4, tableone 0.7.10 [25], scipy 1.7.1, NumPy 1.20.3, seaborn 0.11.2, matplotlib 3.4.3, sklearn 0.24.2 and statsmodels 0.12.2. The normality of data was assessed with the Shapiro–Wilk test. The continuous variables that followed a normal distribution were presented as mean ± standard deviation (SD); meanwhile, those that did not follow a normal distribution were presented as median and interquartile range. Categorical variables were in actual numbers and percentages.

Spearman correlation was used for discrete continuous variables and Bonferroni correction for multiple comparisons. Scores were compared using Student's t for two samples for normal continuous variables, the Mann–Whitney U test for non-normal continuous variables, and Fisher's exact test for categorical variables.

Linear multivariate models were performed using Ordinary Least Squares, in which demographic variables that could act as a confounder and variables that showed a correlation in Spearman's test were included. Models better fit a lower Bayesian Information Criterion value were selected.

Receiver operating characteristic (ROC) curves were used to assess the usefulness of the P300 wave amplitude and latency scores in predicting a worse cognitive performance. We assessed discrimination by calculating the area under the ROC curve of sensitivity versus one minus the specificity. An area of 1 implies a test with perfect sensitivity and specificity, while an area of 0.5 implies that the model's predictions are no better than chance.

We calculated a sample size of 25 patients according to the population mean of the latency of the P300 wave (346.9 ms; SD = 38.1 ms) and a margin of error of 15 ms.

## Results

The baseline characteristics (age, sex, and education) and the results of the cognitive tests of the patients are shown in Table 1. The mean age of the patients (11, 36.7% women) was 36.6 years (SD 11.9). Nine (30%) had a hemispherical lesion.

**Table 1:** Baseline characteristics of the patients (*N* = 30)

Age, mean (SD)		36.6 (11.9)
Sex, *n* (%)	Women	11 (36.7)
	Men	19 (63.3)
Location of the cerebellar lesion, *n* (%)	Hemispheric	9 (30.0)
	Pancerebellar	21 (70.0)
Years of education, median [Q1, Q3]		8.0 [6.0, 10.0]
ICARS posture, mean (SD)		10.5 (5.4)
ICARS kinetic, median [Q1, Q3]		13.0 [9.0, 15.0]
ICARS speech, median [Q1, Q3]		3.0 [2.0, 4.0]
ICARS eye movement, mean (SD)		2.6 (1.3)
ICARS total, mean (SD)		28.4 (9.6)
Serial subtraction, median [Q1, Q3]		4.0 [3.0, 5.0]
Backward Digit Span, median [Q1, Q3]		3.0 [2.0, 3.8]
Category Fluency, median [Q1, Q3]		12.0 [8.0, 15.8]
Phonemic fluency, mean (SD)		7.3 (3.0)
Immediate Recall Trial 1, mean (SD)		4.2 (1.1)
Immediate Recall Trial 2, median [Q1, Q3]		5.0 [4.0, 6.0]
Immediate Recall Trial 3, mean (SD)		5.7 (1.4)
Delayed recall, median [Q1, Q3]		6.0 [4.0, 6.0]
Recognition, mean (SD)		14.3 (2.6)
Construction, mean (SD)		8.6 (3.1)
FAB Score, mean (SD)		13.9 (3.0)
Motor Luria, *n* (%)	Impaired	20 (66.7)
	Normal	10 (33.3)
Graphic Luria, *n* (%)	Impaired	21 (70.0)
	Normal	9 (30.0)
P300 Latency, mean (SD)		366.8 (40.1)
P300 Amplitude, mean (SD)		7.6 (2.8)

Mean (median) ± standard deviation (SD) and frequency (%) are reported. Median and interquartile ranges were reported if the variables did not follow a normal distribution

The cognitive performance of our sample was below the normative data for the Indian population [26] in serial subtraction [3.63 (SD 1.3) vs.4.8 (SD 0.6); *p* < 0.001], total immediate recall [14.8 (SD 3.32) vs. 16.79 (SD 3.78); *p* = 0.004] and recognition [14.30 (SD 2.58) vs. 18.73 (SD 2.05); *p* < 0.001]. On the contrary, they performed better than expected according to the normative data for phonemic fluency [7.27 (SD 2.99) vs. 5.53 (SD 3.84); *p* = 0.013]. Category fluency [11.67 (SD 3.48) vs. 12.11 (SD 4.7); *p* = 0.610], delayed recall [5.23 (SD 1.43) vs. 4.99 (SD 1.84); *p* = 0.478] and construction tasks [8.57 (SD 3.07) vs. 9.59 (SD 3.27); *p* = 0.087] showed no statistically significant differences. The motor Luria maneuver was altered in 20 (66.7%) patients, and the graphic Luria maneuver in 21 (70%). Overall, they obtained an average FAB score of 13.9 (SD 2.98), with nine (30%) patients having a result below 12 points. Women performed better than men in construction tasks [10.3 (SD 1.8) vs. 7.6 (SD 3.2); *p* = 0.007], but all the rest of the tests did not show significant differences.

The P300 wave values showed an increase in latency to normative population data [366.83 ms (SD 40.11 ms) vs. 346.9 ms (SD 38.1 ms); *p* = 0.004] and a lower amplitude with a non-significant trend [7.56 ms (SD 2.79 ms) vs. 9.2 ms (SD 5.0 ms); *p* = 0.072]. Women showed a greater amplitude than men [8.9 mV (SD 2.6 ms) vs. 6.8 mV (SD 2.6 ms); *p* = 0.038] and a non-significant trend to have a lower latency [352.0 ms (SD 30.0 ms) vs. 375.4 ms (SD 43.4 ms); *p* = 0.094].

**Fig. 1:**
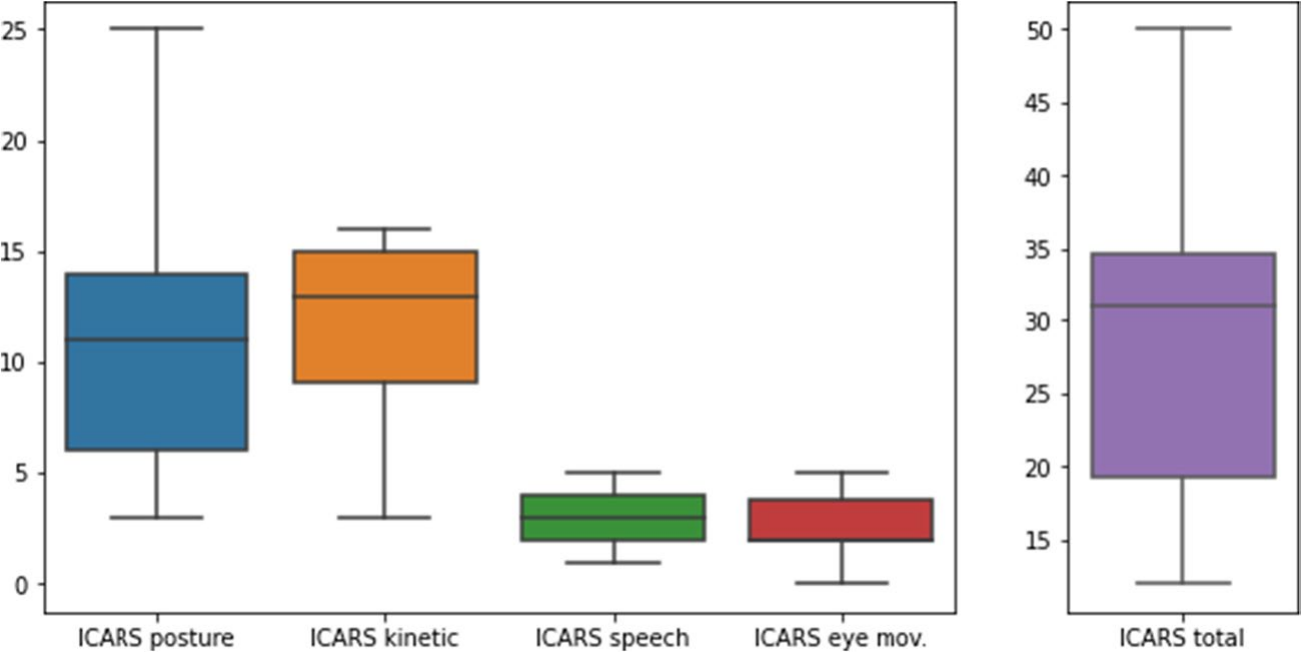
Boxplot showing the distribution of the ICARS and its subscales in our sample. The values of the different subscales, and the total score are displayed

The ICARS score is shown in Fig. 1. The mean score of the total ICARS was 28.37 (SD 9.64); meanwhile, the score in the posture and gait disturbance domain was 10.53 (SD 5.36), and for the kinetic function, 12.00 (SD 3.89). Similarly, the mean score in the speech disorder domain was 3.27 (SD 2.33), and the mean score in the eye movement disorder domain was 2.57 (SD 1.25).

**Fig. 2:**
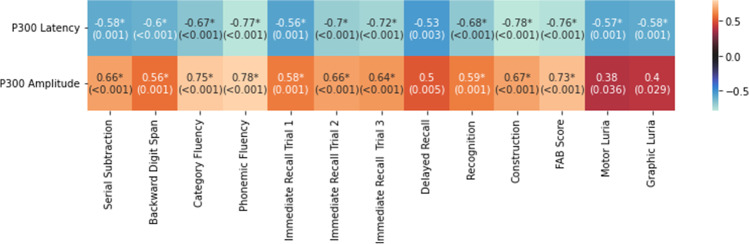
Spearman's r between p300 wave parameters and cognitive tests [r (p-value)]. The values marked with an asterisk reached significance (adjusted for multiple comparisons)

There was a significant negative correlation between the cognitive tests and the p300 wave latency, except for delayed recall (Fig. 2). Likewise, there was a positive correlation between the cognitive tests and the amplitude of the p300 wave, except for the delayed memory and Luria maneuvers, both motor and graphic (Fig. 2).

**Fig. 3:**
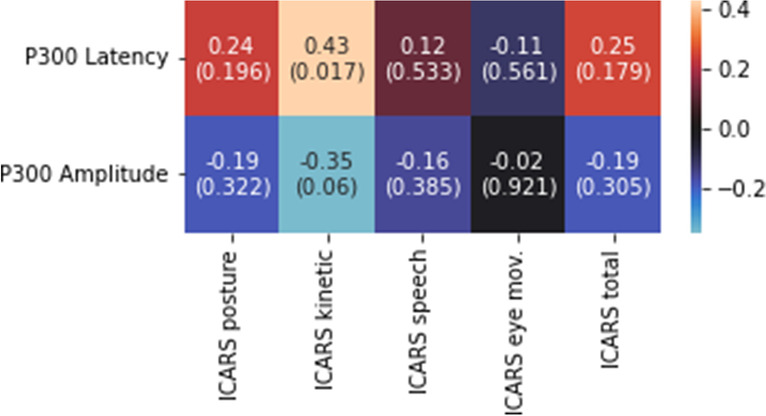
Spearman's r between the parameters of the p300 wave and the ICARS and its subscales [r (p-value)]. The values marked with an asterisk reached significance (adjusted for multiple comparisons)

On the other hand, there was a significant positive correlation between the ICARS kinetic subscale and the latency of the P300 wave (r = 0.43; *p* = 0.017) and a negative trend between the same subscale and the amplitude of the P300 wave (r = -0.35; *p* = 0.06) (Fig. 3). The correlations of the P300 wave amplitude and latency with the ICARS total score and the rest of the subscales were non-significant (Fig. 3).

**Fig. 4:**
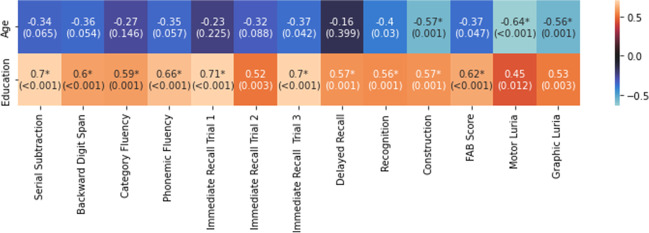
Spearman's r between age, education, and cognitive tests [r (p-value)]. The values marked with an asterisk reached significance (adjusted for multiple comparisons)

Education level was positively correlated with all cognitive tests, as shown in Fig. 4. There was a negative correlation between age and construction tasks (r = -0.57; *p* = 0.001), Luria motor maneuver (r = -0.64; *p* < 0.001), and graphic Luria maneuver (r = -0.56; *p* = 0.001) (Fig. 4). Likewise, there was also a negative correlation between age and Immediate Recall Trial 3 (r = -0.37; *p* = 0.042), recognition (r = -0.4; *p* = 0.03), and the FAB score (r = -0.37; *p* = 0.047) (Fig. 4).

**Fig. 5:**
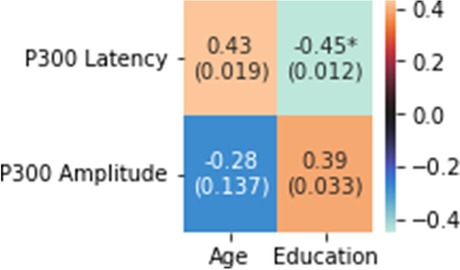
Spearman's r between the age and education and the parameters of the P300 wave [r (p-value)]. The values marked with an asterisk reached significance (adjusted for multiple comparisons)

There was no correlation between the demographic variables and the ICARS total score scale but negative between the latency of P300 and education (r = -0.45; *p* = 0.012) and positive with age (r = 0.43; *p* = 0.019) and between the amplitude of P300 and education (r = -0.39; *p* = 0.033) (Fig. 5).

**Fig. 6:**
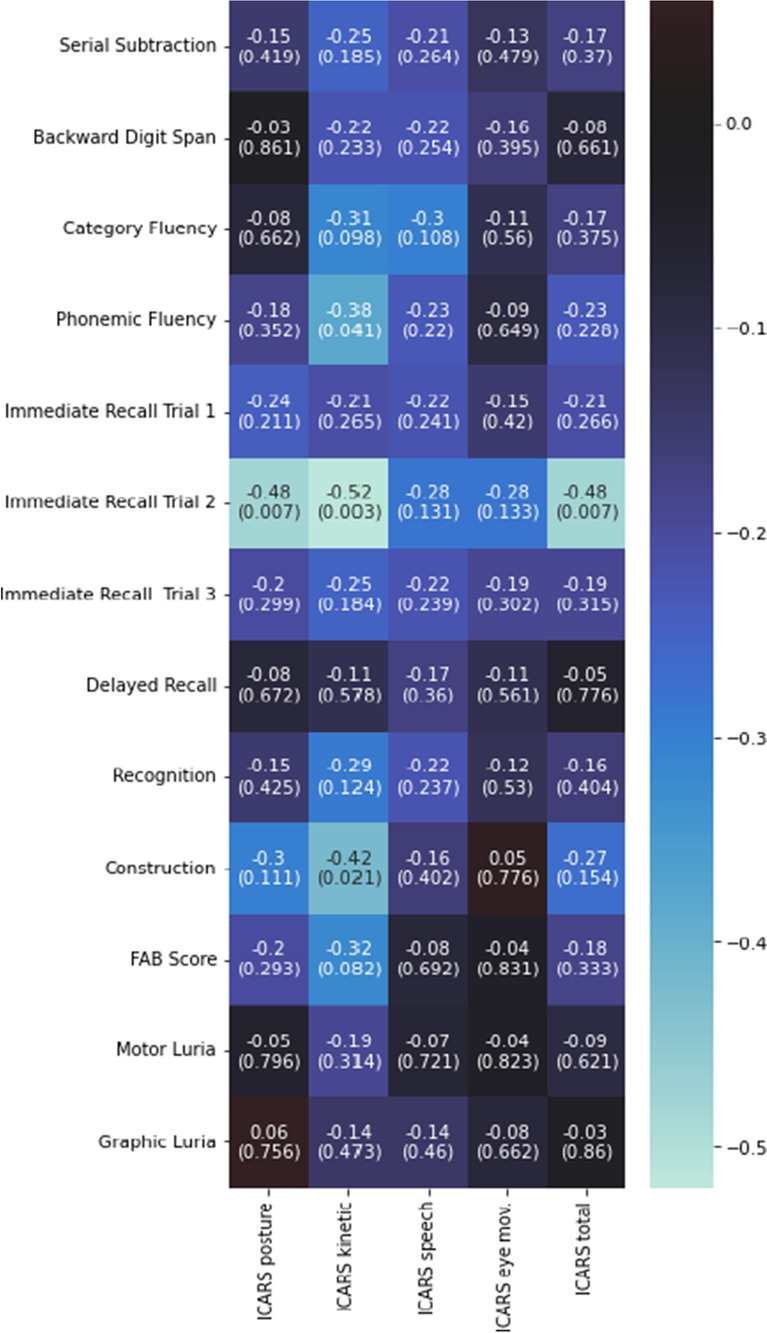
Spearman's r between the ICARS and its subscales and cognitive tests [r (p-value)]. The values marked with an asterisk reached significance (adjusted for multiple comparisons)

Regarding the relationship between the ICARS and cognitive performance, negative correlations between the ICARS kinetic subscale and phonemic fluency (r = -0.376; *p* = 0.041), Immediate Recall Trial 2 (r = -0.52; *p* = 0.003), and construction tasks (r = -0.42; *p* = 0.021) were found. There was also a significant negative correlation between Immediate Recall Trial 2 and the ICARS posture subscale (r = -0.48; *p* = 0.007) and ICARS total score (r = -0.48; *p* = 0.007). The rest of the values are shown in Fig. 6.

When separating patients according to the lesion localization (hemispheric or pancerebellar), there were no differences in latency [353.9 ms (SD 32.0 ms) vs. 372.4 ms (SD 42.6 ms); *p* = 0.206] or amplitude [8.5 mV (SD 2.5 mV) vs. 7.2 mV (SD 2.9 mV); *p* = 0.221] of the P300 wave. There were also no differences in performance in ICARS total score [25.3 (SD 10.1) vs. 29.2 (SD 9.1); *p* = 0.34] or any of its subscales. Likewise, there were no differences in cognitive performance except in delayed recall [6.1 (SD 0.9) vs. 4.9 (SD 1.5); *p* = 0.01], in which patients with hemispheric lesions showed better performance than the normative data.

In a multivariate model, P300 wave latency was positively associated with the ICARS kinetic subscale (*p* = 0.005) and age (*p* = 0.009), regardless of sex and years of education. In the model that included cognitive variables, P300 wave latency was negatively associated with performance in phonemic fluency (*p* = 0.035) and construction (*p* = 0.009) independently of age and education level. On the other hand, P300 wave amplitude was positively associated with the FAB total score (*p* < 0.001) independently of age, sex, and education level. In the case of the model that included the ICARS total score, the kinetic subscale showed an inverse relationship with a non-significant trend (*p* = 0.055) but positive with the female sex (*p* = 0.034).

**Fig. 7:**
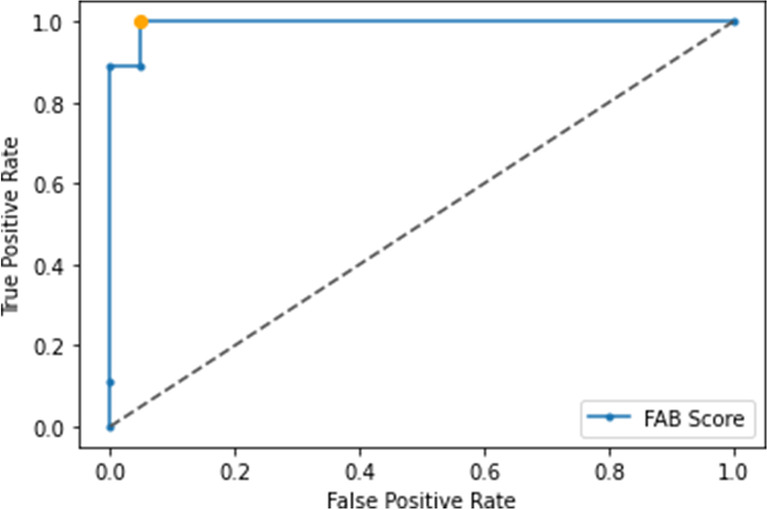
ROC curve for the determination of an altered Frontal Assessment Battery score (AUC = 0.995, threshold 5.81 mV, sensitivity = 1, specificity = 0.95) according to the amplitude of the P300 wave

**Fig. 8:**
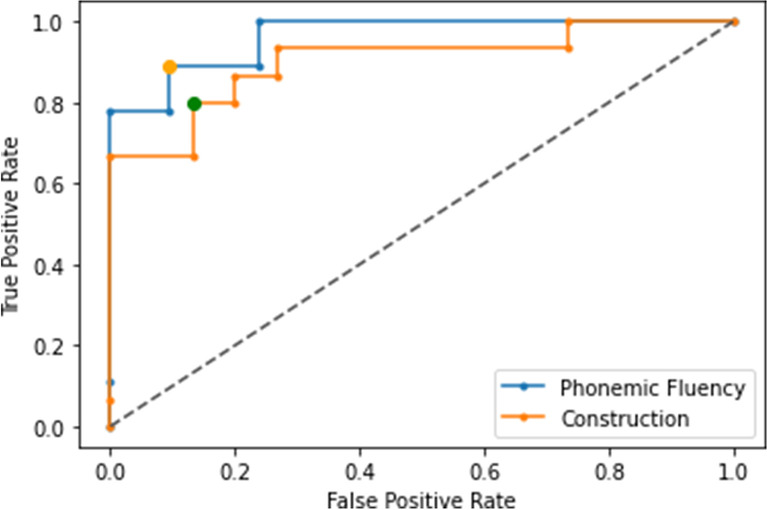
ROC curve for the determination of the worse performance of phonemic fluency (AUC = 0.963, threshold 392.73 ms, sensitivity = 0.88, specificity = 0.90) and construction tasks (AUC = 0.902, threshold 376.36 ms, sensitivity = 0.8, specificity = 0.86) according to the latency of the P300 wave

The area under the ROC curve (AUC) for an altered FAB score was 0.995 for the P300 wave amplitude, with a sensitivity of 1 and a specificity of 0.95 (cut-off point of 5.81 mV). Phonemic fluency had a cut-off point of 392.73 ms (AUC = 0.963) in P300 wave latency with a sensitivity of 0.88 and a specificity of 0.90. Construction tasks had a cut-off point of 376.36 ms (AUC = 0.902) with a sensitivity of 0.8 and a specificity of 0.86. Cut-off point results showed no statistically significant differences from population values [FAB score: pooled z-test = -0.68, *p* = 0.5; phonemic fluency: pooled z-test = 0.77, *p* = 0.44; construction: pooled z-test = 1.2, *p* = 0.23]. These results are shown in Figs. 7 and 8, respectively.

## Discussion

We found that patients with cerebellar lesions showed a statistically significant increase in p300 wave latency [366.83 ms (SD 40.11 ms) vs. 346.9 ms (SD 38.1 ms); *p* = 0.004] with a non-significant decrease in amplitude [7.56 mV (SD 2.79 mV) vs. 9.2 mV (SD 5.0 mV); *p* = 0.072], suggesting that the cerebellum is related to attention [5] as this wave is an indirect measure of attention [27].

The alterations found in the P300 wave agree with previous studies conducted in patients with cerebellar pathology. Studies in SCA-2 [16] or olivopontocerebellar atrophy patients [17] have shown an increase in latency and a lower amplitude than the control group in the first and without data in the second study. Other studies have not found differences in wave or learning performance between patients with cerebellar pathology and healthy controls [28]. However, the wave does react differently to negative and positive conditioning in patients with cerebellar pathology [28].

Additionally, P300 wave alterations were associated with poorer cognitive functioning and performance in specific subscales of ICARS. It is well known that the P300 wave alterations are related to mild cognitive impairment [17, 29, 30], episodic memory [14, 31], working memory [31], and attention [31]. In other studies on Parkinson's disease [32, 33], verbal fluency, visual-spatial perception, and abstract reasoning were also impaired. Nonetheless, to our knowledge, it is the first time it has been related to the ICARS.

In the multivariate model in which we assessed the cognitive variables and the latency of the P300 wave, phonemic fluency and construction tasks were associated with a latency delay independent of age, sex, and education. In univariate analysis, a positive correlation was found between the ICARS kinetic subscale and the latency of the P300 wave. Meanwhile, a negative correlation was found between these two cognitive domains (phonemic fluency and construction tasks) and the ICARS kinetic subscale.

Similar to what we observed, previous studies have found a correlation between the ICARS score and performance on different cognitive tests [34–36]. A study of patients with cerebellar lesions [34] found a correlation between the ICARS and the Trail Making Test, Rey-Osterrieth Complex Figure, FAB, phonemic fluency, and Five-Point Test. In the current study, there was also a correlation between different parts of the tests and the ICARS posture and ICARS speech subscales. Other studies in patients with SCA1, SCA2, and SCA3 negatively correlated the ICARS with working memory, visuospatial perception, executive function [35], and alterations in the Mini-Mental State Examination, anxiety, depression, fatigue, and sleep [36]. The results of these studies show that alterations in cognitive scales are influenced by disability measured by the ICARS in a similar way to what our results show.

The influence of the ICARS on the performance of these tests may depend on cognitive ability and other characteristics of the tests. The construction tasks in our sample were assessed with geometric drawings according to the Kolkata Cognitive Screening Battery scale [21]. These tasks depend not only on manual ability but also on visuospatial capacity, which is, in turn, correlated with an increase in P300 wave latency in other diseases, such as Parkinson's disease [31, 32].

On the other hand, when measuring verbal fluency (semantics and phonemics), there was no clear correlation with the ICARS speech subscale. The explanation for this finding may be that phonemic fluency does not have the same origin as spontaneous language fluency. This finding has been previously reported in other studies showing a correlation between semantic fluency and other cognitive tests but not phonemic fluency [9].

All these findings suggest the ICARS kinetic subscale, phonemic fluency, construction tasks, and P300 wave latency act as confounding factors between them.

Regarding the multivariate model that assessed the relationship of amplitude with cognitive variables, we found that performance in the FAB was positively related to amplitude (*p* < 0.001). None of the models with cerebellar tests reached statistical significance, but the female sex did so positively.

A previous study linked the decrease in amplitude with different frontal lobe function tests, although not with the FAB [37]. In that study, [37] a positive correlation was found between amplitude and the Stroop test, copying tasks, verbal fluency, and similarities. Some of these subscales are present in the FAB, and although the total result did not show significance in that study, [37] it aligns with the results obtained in ours.

All these findings indicate that cerebellar lesions are not as closely related to changes in amplitude as disability measured by the ICARS, suggesting that the cerebellum does not have as much relevance in the amplitude of the P300 wave in the same way that its lesion does not seem to affect executive tasks.

ROC curves were performed to determine the latency cut-off points that predicted lower performance in phonemic fluency (threshold 392.73 ms) or construction tasks (threshold 376.36 ms) and also the cut-off point of the amplitude of the P300 wave that predicted a worse performance in the FAB (threshold 5.81 mV). These three cut-off points showed AUC values close to 1 with sensitivity and specificity above 80%. However, these values were similar to the general population. Hence, they cannot be applied to discriminate which patients have cognitive impairment.

Our study is not without limitations. First, the sample size was relatively small. However, we observed robust associations. Notwithstanding, it would be important to replicate these findings in a larger sample, as small samples may be subject to spurious findings. Second, we did not have a control group, so we compared the obtained data with the population. Third, we recruited hospitalized patients, limiting our external validity. In order to avoid this problem, an attempt to rule out any intercurrent processes that may limit our analysis was performed. Fourth, we did not collect affective data or recruit patients without a history of significant psychiatric pathology. Fifth, though we tried to select patients with lesions restricted to the cerebellum by performing brain MRI and 18F-FDG PET-CT, there is still the possibility of involvement of other brain parts. Finally, our patients had cerebellar pathology of different etiologies with different duration of diseases limiting the generalizability of the results. 

## Conclusion

Patients with cerebellar damage had an increase in latency and a decrease in the amplitude of the P300 wave, which agrees with previous studies [16, 17]. These P300 wave alterations were also associated with worse cognitive performance and some of the subscales of ICARS, reinforcing that the cerebellum has both motor, cognitive, and affective functions [9]. Likewise, these findings indicate that the cerebellum is also related to attention [5] and the salience network [38, 39]. We also found that patients with cerebellar lesions performed worse on several cognitive tests, such as repeated subtractions, phonemic fluency, immediate recall, and recognition. Of note is that several of these tests (repeated subtractions [40], phonemic fluency [41, 42], and immediate recall [43, 44]) are closely related to attention. Furthermore, the results on the ICARS kinetic subscale were most correlated with worse performance on immediate memory, phonemic fluency, and visuospatial tasks and with an increase in the latency of the P300 wave. All these interrelationships reinforce the idea that the cerebellum has functions in attention and cognition [5, 9] and that the severity of the lesion involvement, as measured by the ICARS kinetic subscale, is related to the severity of cognitive impairment.

## Data Availability

The data supporting this study's findings are available from the corresponding author, [J.B.-L.], upon reasonable request.
